# Insights for enhancing participation in dementia research in low‐ and middle‐income countries (LMICs): Voices from the fields in Southwest Nigeria

**DOI:** 10.1002/alz70860_104775

**Published:** 2025-12-23

**Authors:** Olufisayo Oluyinka Elugbadebo, Faithful Moposi Akintade, Cynthia Olapeju Olawuyi, Chiamaka Faith Okwudiri, Faith Ejimkonye, Olusegun Baiyewu

**Affiliations:** ^1^ University College Hospital, Ibadan, Oyo, Nigeria; ^2^ College of Medicine, University of Ibadan., Ibadan, Oyo, Nigeria; ^3^ Institute for Advanced Medical Research and Training, Ibadan, Oyo, Nigeria; ^4^ University of Ibadan, Ibadan, Oyo, Nigeria; ^5^ University College Hospital, Ibadan, Oyo State, Nigeria; ^6^ Department of Psychiatry, University of Ibadan, Ibadan, Oyo, Nigeria; ^7^ Institute for Advanced Medical Research and Training, College of Medicine, University of Ibadan, Ibadan, Nigeria

## Abstract

**Background:**

Engaging participants in research is crucial for advancing scientific knowledge, but recruitment and retention pose significant challenges in studies involving older adults. In low‐ and middle‐income countries (LMICs), unmet expectations regarding study procedures and perceived benefits often result in dissatisfaction and attrition. Despite the growing burden of dementia in LMICs, there is limited understanding of how these factors influence participants' participation in dementia research. This study explores the experiences and perspectives of dementia research participants to identify strategies for improved engagement, retention, and ethical practices in dementia research within LMICs.

**Method:**

A narrative qualitative approach was used to explore participants' experiences with dementia research in Southwest Nigeria. Key informant interviews (KIIs) were conducted with 15 family dyads who had participated in dementia studies in the community or clinic setting between January and September 2024. The interview guide explored participants’ experiences, expectations, perceived impacts, and recommendations for future research. Data were transcribed verbatim into English, where necessary, and analyzed thematically using an inductive approach with NVivo software.

**Result:**

Among the 15 dyads interviewed, 7 males and 8 females were diagnosed with dementia, aged 64–86 years. Participants reported challenges, including long distances to recruitment sites, prolonged interview durations, physically demanding activities, inadequate incentives, and a lack of involvement in research planning. Participants anticipated research benefits such as improved access to healthcare, treatments, and subsidized or free medications during the study; however, these were often unmet. A key source of dissatisfaction was the lack of communication of study findings. Positive aspects highlighted included increased knowledge about dementia, engaging cognitive activities, and improved awareness of healthy lifestyle practices. Participants emphasized the importance of effective dissemination of research findings. Despite the challenges, participants expressed willingness to engage in future research, citing trust from previous engagements with researchers and perceived personal and societal benefits as motivating factors.

**Conclusion:**

This study underscores the critical need to align research practices with a patient participatory approach, ensuring transparency in communication, fair compensation, and effective dissemination of findings. These strategies will not only enhance participant satisfaction and retention but foster ethical, impactful research in resource‐constrained settings.

